# Dendritic Cells are preferentially targeted among hematolymphocytes by Modified Vaccinia Virus Ankara and play a key role in the induction of virus-specific T cell responses *in vivo*

**DOI:** 10.1186/1471-2172-9-15

**Published:** 2008-04-15

**Authors:** Luzheng Liu, Rahul Chavan, Mark B Feinberg

**Affiliations:** 1 Harvard Skin Disease Research Center, Department of Dermatology, Harvard Medical School, Brigham and Women's Hospital, Boston, MA 02115, USA; 2 Emory Vaccine Center, Emory University School of Medicine, Atlanta, GA 30329, USA; 3 University of South Florida College of Medicine, Tampa, FL 33612, USA; 4 Merck Vaccine Division, West Point, PA 19486, USA

## Abstract

**Background:**

Modified Vaccinia Ankara (MVA) is a highly attenuated strain of vaccinia virus (VV) that has lost approximately 15% of the VV genome, along with the ability to replicate in most mammalian cells. It has demonstrated impressive safety and immunogenicity profile in both preclinical and clinical studies, and is being actively explored as a promising vaccine vector for a number of infectious diseases and malignancies. However, little is known about how MVA interacts with the host immune system constituents, especially dendritic cells (DCs), to induce strong immune responses despite its inability to replicate in vivo. Using *in vitro *and *in vivo *murine models, we systematically investigated the susceptibility of murine DCs to MVA infection, and the immunological consequences of the infection.

**Results:**

Our data demonstrate that MVA preferentially infects professional antigen presenting cells, especially DCs, among all the subsets of hematolymphoid cells. In contrast to the reported blockage of DC maturation and function upon VV infection, DCs infected by MVA undergo phenotypic maturation and produce innate cytokine IFN-α within 18 h of infection. Substantial apoptosis of MVA-infected DCs occurs after 12 h following infection and the apoptotic DCs are readily phagocytosed by uninfected DCs. Using MHC class I – deficient mice, we showed that both direct and cross-presentation of viral Ags are likely to be involved in generating viral-specific CD8^+ ^T cell responses. Finally, DC depletion abrogated the T cell activation *in vivo*.

**Conclusion:**

We present the first *in vivo *evidence that among hematolymphoid cells, DCs are the most susceptible targets for MVA infection, and DC-mediated Ag presentation is required for the induction of MVA-specific immune responses. These results provide important information concerning the mechanisms by which strong immune responses are elicited to MVA-encoded antigens and may inform efforts to further improve the immunogenicity of this already promising vaccine vector.

## Background

The success of the prototype poxvirus vaccinia virus (VV) to eradicate naturally occurring smallpox worldwide stands as one of the most remarkable medical achievements in human history [1]. This achievement came at a price, however. Inoculation with live VV can overwhelm the immune system of immunocompromised individuals, causing significant morbidity and mortality [2,3]. As a result of this realization, toward the end of the smallpox immunization campaign, a highly attenuated VV strain, modified vaccinia virus Ankara (MVA), was developed for use in individuals at high risk of VV-associated adverse events. MVA has since been safely administrated to over 120,000 individuals in the late stages of the smallpox eradication effort [4]. MVA lost approximately 15% of the vaccinia genome in the course of over 500 passages in chicken embryonic fibroblasts *ex vivo*, along with the ability to replicate in most primary mammalian cells [4,5]. Compared to replicating VV, MVA provides similar or higher levels of endogenous or recombinant gene expression even in non-permissive cell lines, higher but more transient Ag expression *in vivo*, and comparable levels of immune responses in animal models [6-12]. In light of this favorable degree of immunogenicity, combined with its highly attenuated phenotype and associated inherent safety properties, MVA is being actively explored as a promising vaccine vector in both preventative and therapeutic indications for a number of infectious diseases and malignancies. In addition, MVA has also been the subject of renewed interest and clinical evaluation as a safer vaccine to prevent smallpox in light of concerns about potential bioterrorist threats and the increased sensitivity to vaccine safety considerations especially at a time when far more individuals with acquired immunodeficiency (by virtue of either therapeutic immunosuppression or HIV infection) are present in the population than when the smallpox eradication campaign was mounted [11,13,14].

Murine models represent an extensively utilized preclinical experimental system for vaccine development. In the case of MVA, the murine model has been employed, to date, to study both MVA-induced cellular and humoral immune responses, as well as the efficacy of protection following experimental pathogenic VV challenge [15,16]. However, little has been done to understand how this highly attenuated non-replicating virus activates both branches of the immune system. Of particular interest, few systematic studies have been carried out to investigate the interaction between MVA and murine dendritic cells (DCs), the most potent APCs to activate naïve CD4 and CD8 T cells [17]. In contrast, there has been considerable interest and effort invested in the study of the interaction of VV and human DCs, and to a somewhat lesser extent, the interaction between MVA and human DCs [12,18]. VV has been shown to abortively infect human DCs, block their maturation, inhibit their phagocytosis and directional migration, and to induce extensive DCs apoptosis [19-22]. These observations suggest that targeted infection of human DCs by VV may be an important viral strategy to circumvent host immune defenses. All of these studies employed human monocyte-derived DC (MoDC) cultures. It has not been assessed, in the very tractable murine model system, how and to what extent specific consequences of poxvirus infection of DC manifested in culture predict the ability of the host to generate antiviral immune responses *in vivo*. Since MVA has lost multiple host range genes as well as immunomodulatory genes present in the parental VV genome [5,23], it is conceivable that the target cell tropism of MVA may differ from that of VV, and MVA might activate instead of suppress the maturation and function of DC, as suggested for VV.

In the current study, we sought to investigate the following questions in murine models: (1) the susceptibility of DCs to MVA infection; (2) the phenotypic and functional alteration of DCs upon MVA infection; (3) whether DCs activate T cells via direct or cross-presentation, and (4) whether DCs are required for *in vivo *T cell priming following MVA infection. Knowledge gained from these studies can provide important information concerning the mechanisms by which strong immune responses are elicited to MVA-encoded antigens.

## Results

### Murine professional APCs are preferentially targeted by MVA among hematolymphoid cells, with DCs being the most susceptible targets

**Figure 1:**
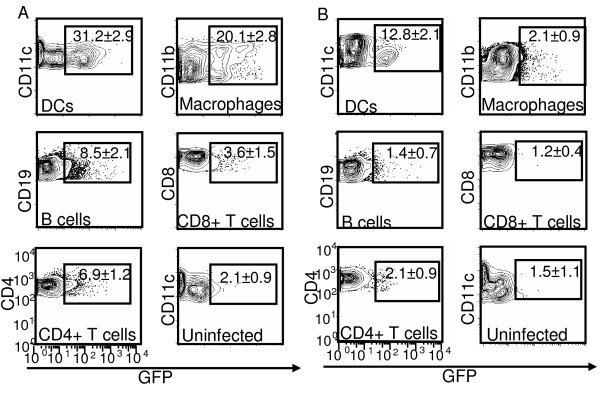
**MVA preferentially targeted professional APCs, especially DCs, both *in vitro *and *in vivo***. (A) Splenocytes from naïve BALB/c mice were infected with rMVA-GFP at a MOI of 10 *in vitro *for 8 h followed by cell surface marker staining and flow cytometry analysis. Uninfected splenocytes were used as negative control. (B) BALB/c mice were either uninfected or infected with rMVA-GFP at 3 × 10^8 ^PFU/mouse by i.v. injection. Spleens were harvested at 9 h post infection and GFP expression in various subsets of splenocytes was monitored by flow cytometry. DCs: CD11c^+^; macrophages: CD1b^+^CD11c^-^; B cells: CD19^+^; CD8^+ ^T cells: CD8^+ ^CD11c^-^; CD4^+ ^T cells: CD4^+ ^CD11c^-^. Numbers shown are the percentages (average ± standard deviation (SD)) of GFP^+ ^cells in the corresponding cell subsets. The data are representative of three independent experiments.

To study whether murine DCs are susceptible to MVA infection, naïve BALB/c splenocytes were infected *in vitro *with rMVA-GFP at a MOI of 10 for 8 h followed by flow cytometric detection of EGFP expression in different cell populations. As shown in Fig. 1A, despite the presumed broad cellular tropism of VV and related viruses, rMVA-GFP did not infect all subsets of splenocytes to an equivalent extent, but rather demonstrated a striking preference for professional APC populations, with CD11c^+ ^DCs being the most susceptible targets for MVA infection in the spleen (31.2 ± 2.9% GFP^+^), followed by CD11b^+ ^CD11c^- ^macrophages (20.1 ± 2.8% GFP^+^) and CD19^+ ^B cells (8.5 ± 2.1% GFP^+^). Very few GFP^+ ^cells were detected in CD4^+ ^(6.9 ± 1.2%) and CD8^+ ^(3.6 ± 1.5%) T cell populations. Further phenotypic analysis revealed that over 50% of all GFP^+ ^cells were CD11c^+ ^DCs (data not shown). Therefore, professional APCs, especially DCs and macrophages, seem to be preferentially targeted by MVA among all the splenocytes subsets in the *in vitro *infection setting. This was held true when splenocytes were infected at a range of MOIs (from 0.3 to 10) (data not shown). At all the MOIs tested, DCs were by far the most susceptible targets among all the splenocytes subsets, followed by macrophages and B cells.

To confirm the cellular tropism of MVA infection *in vivo*, we infected BALB/c mice intravenously with 3 × 10^8 ^PFU rMVA-GFP. At various time points post infection, spleens were harvested and MVA infected GFP^+ ^splenocytes were identified by cell lineage marker staining and flow cytometry. The number of GFP^+ ^cells in spleen peaked at 9 h post infection and rapidly declined to undetectable level by 24 h post infection (data not shown). At 9 h post infection, GFP expression was primarily detected in CD11c^+ ^DC population (12.8 ± 2.1% GFP^+^), but not any other cell types including macrophages, as shown in Fig. 1B. This result suggests that MVA preferentially infects DCs in secondary lymphoid organ *in vivo*. Since DCs are vastly outnumbered by B cells, T cells in spleen, this result, together with the *in vitro *infection data (Fig. 1A), establish that among various subsets of hematolymphoid cells, DCs are preferentially targeted by MVA in a highly effective manner.

### MVA abortively infect murine DCs, with viral life cycle arrested before late gene expression

**Figure 2:**
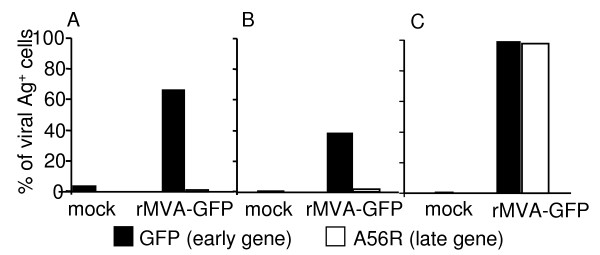
**Only early but not late MVA genes were expressed from infected DCs**. BMDCs (A), splenic CD11c^+ ^DCs (B), and DF-1 cells (C) were infected with rMVA-GFP at a MOI of 10. Mock-treated cells were included as negative controls. MVA-encoded early gene (GFP) and late gene (A56R) expression was monitored 20 h post infection by flow cytometry. The data shown are representative of three independent experiments.

MVA has lost its ability to replicate in most primary mammalian cells. The defect in the viral life cycle has been shown to be the final steps of viral morphogenesis, with no alteration in early or late virus gene expression [6]. To determine whether both early and late MVA genes were expressed from the infected murine DCs, we measured the expression of GFP (driven by an early promoter) and a late viral gene A56R from the *in vitro *MVA-infected BMDCs as well as purified CD11c^+ ^splenic DCs, as described in Methods. The permissive chicken embryonic fibroblasts DF-1 cells were infected in parallel as positive controls for MVA gene expression. Cells were infected with rMVA-GFP at a MOI of 10 for 20 h. The cells were then harvested and subjected to intracellular staining for anti-A56R mAb, as described in Methods. As shown in Fig. 2, the expression of the early gene GFP was easily detected in over 60% of cultured BMDCs and 40% of splenic DCs. However, no A56R (late gene) expression was detected in either DC preparations (Fig. 2A&B), while almost 100% DF-1 cells expressed both GFP and A56R. Therefore, the MVA life cycle is blocked in murine DC at an earlier stage than the virion assembly defect reported for other non-permissive mammalian cell lines [6,12]. These data are consistent with the report that only early but not late MVA genes are expressed from the infected human DCs [12,24].

### Upon MVA infection, BMDC underwent rapid maturation and produced significant amount of IFN-α

**Figure 3:**
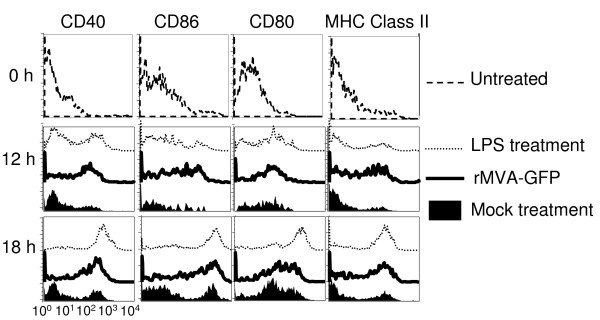
**MVA infection induced rapid BMDC maturation**. Immature BMDCs were mock treated, infected with rMVA-GFP at a MOI of 10, or stimulated with 10 μg/ml LPS as described in Methods. At 0, 12 and 18 h post treatment, cells were harvested and subjected to cell surface staining for the indicated maturation markers. Samples were analyzed by flow cytometry. Histograms were gated on GFP^+ ^cells. These data are representative of five independent experiments.

Targeted infection of MoDC by VV has been suggested as a viral immune evasion strategy, as DC maturation was shown to be blocked by VV infection [20,24]. To study whether MVA infection affects the maturation process of murine DC, we monitored the expression of maturation markers by DCs following rMVA-GFP infection. Initially splenic DCs were used in the experiments. However, these cells underwent rapid apoptosis within 12 h following *in vitro *isolation, even without infection (data not shown). BMDCs were then used to study the effect of MVA infection of DC maturation. BMDCs were infected with rMVA-GFP at a MOI of 10, mock-treated or stimulated with LPS. Mock-treated BMDCs were used to reflect the spontaneous BMDC maturation induced by the "mechanical" manipulation. LPS treatment was included to induce maximal BMDC maturation. At 0, 12 and 18 h following infection, cells were harvested and stained with mAbs specific for CD40, CD86, CD80, and MHC class II I-A^d^. As shown in Fig. 3, as early as 12 h post infection there was a significant upregulation of all the maturation markers on the majority of infected BMDCs. By 18 h post infection, the virus-infected DCs further upregulated the maturation markers. The phenotype of the MVA-infected DCs was comparable to that of LPS-stimulated DCs at both 12 h and 18 h post infection. These data suggest that MVA infection induced rapid BMDC maturation. This is in direct contrast to the reported blockage of human DC maturation by VV infection [19,20,24].

**Figure 4:**
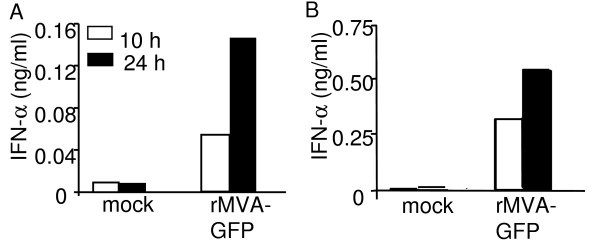
**MVA infection induced IFN-αproduction from DCs**. Immature BMDCs (A) or splenic CD11c^+ ^DCs (B) were either mock treated or infected with rMVA-GFP at a MOI of 10. Supernatant samples were collected at 10 h and 24 h post infection for IFN-α detection by ELISA. These data are representative of three independent experiments.

Activated DCs secrete an array of cytokines and chemokines that are crucial for amplification of both innate and adaptive immune responses [17]. To test whether MVA infection of murine DCs could induce cytokine production, we measured the secretion of the pro-inflammatory cytokine IFN-α from DCs following MVA infection. BMDCs and splenic CD11c^+ ^DCs were either mock-treated or infected with rMVA-GFP at a MOI of 10. Supernatants were harvested at 10 and 24 h post infection, and IFN-α levels were measured by ELISA. As shown in Fig. 4, substantial amount of IFN-α was readily detected from both BMDC and splenic DCs as early as 10 h after rMVA-GFP infection. The amount of IFN-α detected in the supernatant samples was further increased by 24 h post infection. These results suggest MVA-infected DCs not only undergo rapid phenotypic maturation, but also are capable of prompting host immune responses via pro-inflammatory cytokine production. Noticeably, endogenous splenic DCs produced significantly higher amount of IFN-α upon MVA infection compared to BMDCs (5-fold and 3-fold difference at 10 h and 24 h, respectively).

### DCs underwent substantial apoptosis following MVA infection and were phagocytosed by uninfected DCs

**Figure 5:**
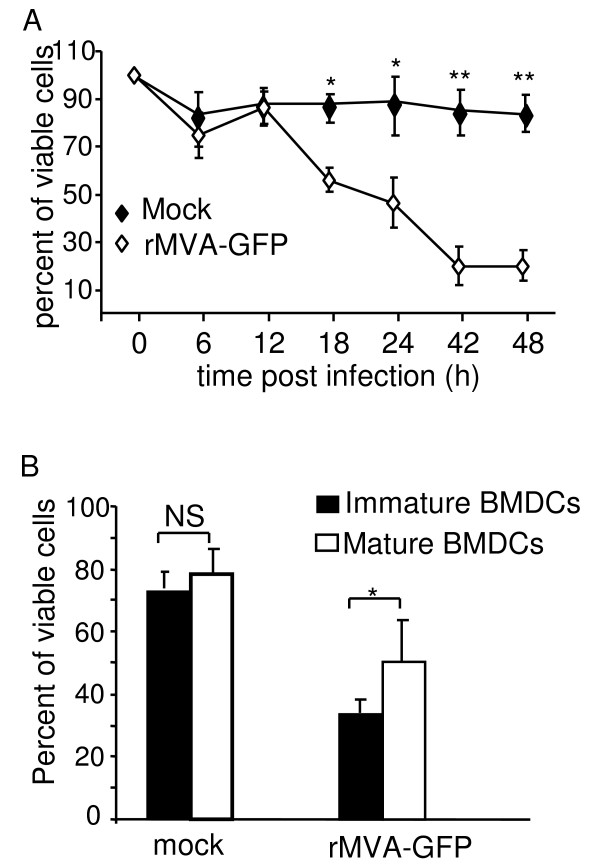
**MVA infection significantly reduced the viability of BMDCs after 12 h of infection**. (A) Kinetic analysis of DC viability following MVA infection. Immature BMDCs were either mock treated, or infected with rMVA-GFP at a MOI of 10. At various time points, cells were harvested and viable cells were counted with trypan blue exclusion method. These data represent the average ± SD viability of 6 replicates from 2 experiments. (B) Immature BMDCs and LPS-stimulated mature BMDCs were mock treated or infected with rMVA-GFP at a MOI of 10. Twenty four hours later, cell viability was examined by trypan blue exclusion. These data represent the average (± SD) viability of 15 replicates from 5 experiments. NS: statistically non-significant; *: P < 0.05; **: P < 0.01.

Studies of VV infection of human MoDCs have suggested that DC undergo substantial apoptosis rapidly following the infection [12,20]. To study whether this is also true for MVA infected murine DC, we next assessed the viability of BMDCs at different time points following MVA infection. Immature BMDCs were either infected with rMVA-GFP at a MOI of 10 or mock treated. Cells were harvested at various time points following the treatment for viability count. As shown in Fig. 5A, immature BMDCs underwent substantial apoptosis starting 12 h following MVA infection, earlier than VV-infected BMDCs which did not undergo significant apoptosis until 18 h post infection (unpublished results). By 24 h following infection, only 50% of the MVA-infected cells remained viable, while 90% of mock-treated cells were still alive. Over 70% of the infected cell had been dead by 42 h following MVA infection. We further compared the viability of immature and mature BMDCs following rMVA-GFP infection. BMDCs were stimulated with LPS for 24 h to reach maturation. Both immature and mature BMDC were then infected with rMVA-GFP or mock-treated for 24 h and the cell viability was determined. As shown in Fig. 5B, MVA infection reduced the viability of both immature and mature BMDC compared to mock treatment. Interestingly, mature BMDCs displayed higher viability than immature BMDCs at this time point. This data suggests that upon maturation, DCs become somewhat more resistant to MVA-induced cell death. This may be of particular importance for direct antigen presentation.

**Figure 6:**
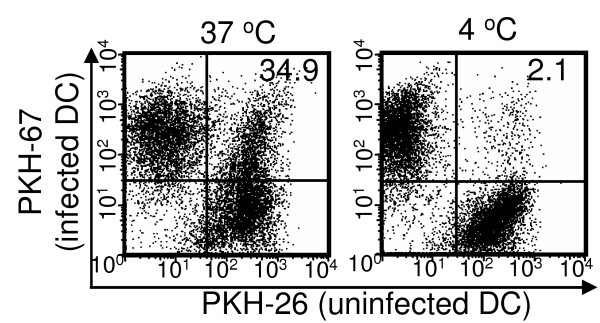
**MVA-infected DCs were phagocytosed by uninfected DCs**. Immature BMDCs were labeled with PKH-67 and infected with MVA at a MOI of 10 for 18 h. They were then extensively washed and mixed with PKH-26-labeled uninfected immature BMDCs for 4 h at either 37 C° or 4 C°. Phagocytosis of MVA-infected DCs by uninfected DCs were detected by flow cytometry. This data is representative of two independent experiments.

The rapid apoptosis of MVA-infected DCs indicates substantial proportion of infected DCs may not present Ags to T cells directly, but rather be uptaken by uninfected bystander DCs for cross-presentation of the viral Ags. We then investigated whether the MVA- infected DCs undergoing apoptosis could be phagocytosed by uninfected DCs. Green fluorescence (PKH-67)-labeled BMDCs were first infected with rMVA for 18 h, then incubated with red fluorescence (PKH-26)-labeled uninfected BMDCs at 1:1 ratio at either 37°C or 4°C for 4 h. Phagocytosis were monitored by flow cytometry. Following incubation at 37°C, one third of the uninfected DCs (30.1 ± 5.3%) became PKH-67^+ ^PKH-26^+^, while few (2.1 ± 1.9%) uninfected DCs picked up PKH-67^+ ^cells at 4°C (Fig. 6). These data suggest that following MVA infection, the infected DCs can be engulfed by uninfected DCs efficiently and rapidly.

### MVA-infected DCs can induce viral antigen-specific CTL activity in vivo by both direct antigen presentation and cross-priming

**Figure 7:**
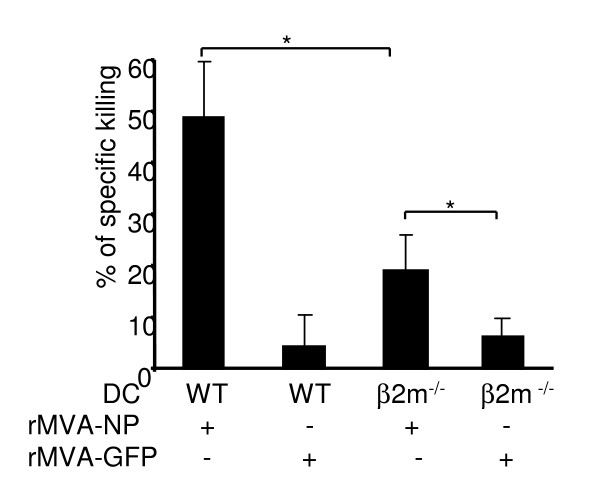
**MVA-infected DCs induced Ag-specific CTL *in vivo via *both direct Ag presentation and cross-priming**. BMDCs derived from either WT C57BL/6 (WT DCs) or MHC class I-deficient β2m^-/- ^mice (β2m^-/- ^DCs) were infected with rMVA-NP or control rMVA-GFP at a MOI of 10 for 6 h. The cells were washed and UV-irradiated to remove and inactivate any residual viruses. DCs were then intravenously injected into LCMV immune C57BL/6 mice 1 × (10^6^). Five days later, NP_396–404_-specific CTL activity in spleens was assessed by fluorescent cellular cytotoxicity assay, as described in Methods. *: P < 0.05.

Having shown that MVA-infected DCs were readily phagocytosed by uninfected bystander DCs, we then hypothesized that MVA Ags can be presented to T cells through cross-presentation. To test this hypothesis, rMVA expressing LCMV NP Ag (rMVA-NP) or rMVA-GFP was used to infect BMDCs from either WT C57BL/6 mice or MHC class I deficient β2m^-/- ^mice. DCs were infected at a MOI of 10 for 6 h before intravenously injected into LCMV immune mice. To prevent direct infection of host APCs by the infectious viruses co-injected with BMDCs, DCs were extensively washed and irradiated prior to the injection. Five days following DC injection, spleens were harvested and single cell suspensions were prepared and CTL activity specific to the dominant LCMV epitope NP_396–404 _was determined by fluorescent cellular cytotoxicity assay, as described in Methods. Strong NP_396–404 _– specific CTL activity was detected in mice immunized by rMVA-NP-infected WT DCs, but not in mice immunized with rMVA-GFP-infected BMDCs (Fig. 7). Interestingly, NP_396–404_-specific CTL activity was also detectable, although to a lesser degree, in mice immunized with rMVA-NP-infectedβ2m^-/- ^DCs. Since β2m^-/- ^DCs cannot directly present MHC class I-restricted Ags to T cells, these data suggest that the detected CTL activity is due to cross-presentation of NP Ags by the endogenous APCs that have phagocytosed the injected β2m^-/- ^DCs.

### *In vivo *DC depletion abrogated the production of effector cytokine IFN-γ by T cells following MVA infection

**Figure 8:**
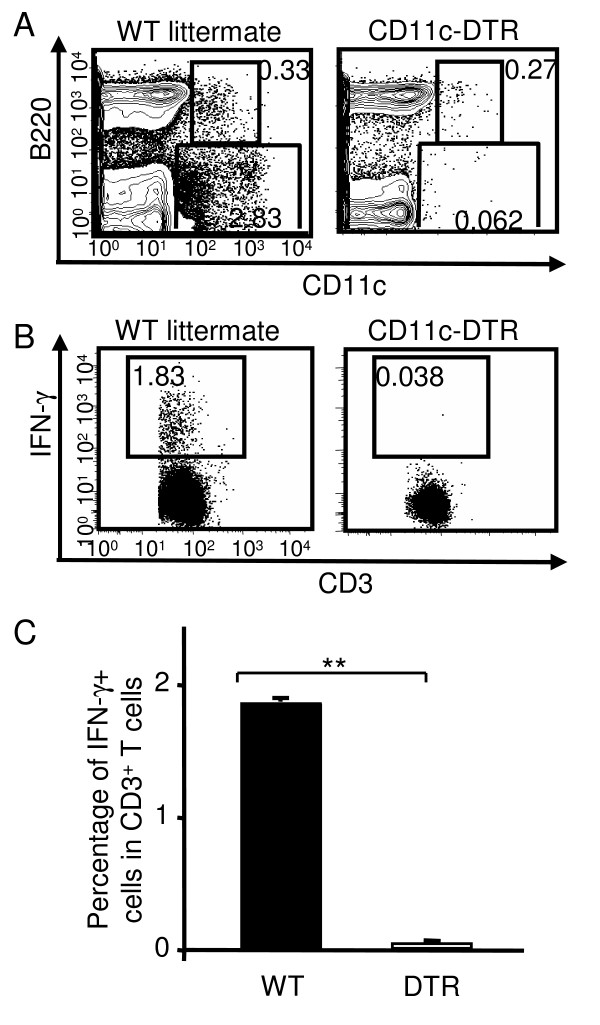
**DCs were required for the generation of IFN-γ-producing effector T cells following MVA infection**. CD11c-DTR mice were depleted of DC by i.p. injection of DT (12ng/g body weight). Six hours after DT treatment, mice were i.p. infected with 5 × 10^6 ^PFU MVA. DT treatment was repeated on day 2 post infection. WT littermates were included as controls. At day 6 following the infection, spleens were harvested to prepare single cell suspension. (A) The efficiency of DC depletion was determined by surface cell lineage marker staining and flow cytometry. Numbers were the percentages of the gated population in total viable splenocytes. (B, C) Spleen single cell suspension (effector) was re-stimulated with MVA-infected C57BL/6 splenocytes (target) for 12 h in the presence of Brefeldin A. Following incubation, the frequencies of IFN-γ-producing T cells in the effector splenocytes were determined by intracellular cytokine staining, as described in Methods. Numbers were the percentages of IFN-γ^+ ^cells in CD3^+ ^T cell population. (C) The average ± SD percentages of IFN-γ^+ ^cells for each group (n = 4). Data are representative of two independent experiments. **: P < 0.01.

To definitively answer the question whether DCs are responsible and required for the *in vivo *T cell activation following MVA infection, we took advantage of the CD11c-DTR mice that express human DT receptor under the control of the CD11c [25]. DT injection has been shown to effectively deplete CD11c^+ ^DCs within 24 h. We depleted DCs from CD11c-DTR mice by i.p. injection of DT (12 ng/g body weight) [25,26] and infected the mice with 5 × 10^6 ^PFU MVA intraperitoneally 18 h later. DT injection was repeated on day 2 after infection to ensure continuous absence of DC. On day 6 after MVA infection, spleens were harvested and the efficiency of DC depletion was determined by flow cytometry. Over 95% of CD11c^+ ^DCs were depleted and the residual DCs had CD11c^low ^B220^+ ^phenotype, similar to that of plasmacytoid DCs (Fig. 8A). Single cell suspensions prepared from the spleens were re-stimulated with C57BL/6 splenocytes infected with MVA for 6 h in the presence of Brefeldin A. Following the re-stimulation, the frequencies of IFN-γ-producing effector CD3^+ ^T cells specific for MVA antigens were determined by intracellular IFN-γ-staining, as described in Methods. As shown in Fig. 8B, MVA-specific effector T cells producing IFN-γ were detected in the spleens of WT littermates on day 6 after MVA infection. These cells accounted for 1.75 ± 0.05% of total CD3^+ ^T cells (Fig. 8C). However, in DC-depleted CD11c-DTR mice, we failed to detect any IFN-γ-production from CD3^+ ^T cells (Fig. 8B and 8C). These data suggest that DC-mediated Ag presentation is required for the generation of IFN-γ producing effector T cells *in vivo *following MVA infection.

## Discussion

rVVs are established tools for the development of vaccines against a wide range of infectious and malignant diseases [15,16]. Concerns about the safety of VV have led to the use of replication-defective viral vector systems, of which MVA is considered one of the safest and most promising candidates. Currently, at least three types of approaches have been used in MVA-based vaccine development [11]: (1) administration of MVA as primary vaccine or as a priming vector in the case of rMVAs expressing heterologous antigens; (2) administration of rMVA as booster vaccine following a primary immunization with an alternative vector encoding the same antigen of interest, such as delivered via DNA vaccination; and (3) immunization with DCs infected *ex vivo *with rMVA. Immunogenicity studies have shown that, compared to the replicating conventional VV vaccines, MVA vaccines can achieve similar or even higher levels of cellular and humoral responses and protect animals from lethal poxvirus challenge [6-12,27]. Although the mechanisms by which this highly attenuated non-replicating virus induces strong immune responses is not well-understood, it is conceivable that all these approaches ultimately depend on the capacity of DCs to induce Ag-specific T cell responses – given the central role of DCs in generating innate and adaptive immune responses. This is supported by the recent report that CD11c^+ ^DCs isolated from Peyer's Patches and spleens of both MVA and VV-immunized mice induced substantial IFN-γ production from an antigen-specific CTL line [27], although whether cell subsets other than DCs from the same lymphoid tissues could also be loaded with Ags and activate T cells was not tested in the study.

Current knowledge of the interaction of poxviruses with DCs is mostly derived from the studies of VV infection of human DC cultures. These studies have shown that VV abortively infects human MoDCs, inhibits their maturation, phagocytosis, and migration, and reduces the ability to stimulate allogeneic or autologous T cells *in vitro *[19-22,24,28]. The suggestion that the perturbation of DC physiology observed in tissue culture infections of human DCs reflect a fundamental strategy of viral immune evasion is difficult to reconcile with the substantial humoral and cellular immune responses generated in VV-vaccinated individuals [29-31]. Whether the results from the *in vitro *studies accurately depict the *in vivo *VV-DC interaction is still an open question. Conflicting data have recently been reported regarding MVA infection of DCs. In one study, MVA was shown to induce immature human MoDC activation, based on the upregulation of co-stimulatory molecules and the secretion of pro-inflammatory cytokines [18]. Another study showed that MVA-infected immature human MoDCs failed to undergo maturation, but nevertheless were able to present viral Ags to the Ag-specific human CTL line [12]. Using a murine model, Belyakov et al showed that endogenous DCs isolated from MVA immunized mice stimulated murine CTL lines *in vitro *[27]. However, the CTL lines used in these studies may have less stringent requirement than primary naïve T cells for co-stimulatory signals provided by APCs. Priming of naïve CD8^+ ^T cells in mice immunized with MVA-infected immature BMDCs was demonstrated by Behboudi et al, even though the infected BMDCs downregulated MHC class I molecules and underwent apoptosis [32,33]. It was unclear in these studies whether MVA Ags were presented directly by MVA- infected DCs or indirectly via cross-presentation in these studies.

In the current report, we systematically characterized the interaction of MVA and host DCs using both *in vitro *and *in vivo *murine models to understand the role of DCs in MVA-induced immunogenicity. Using two different approaches, *in vitro *infection of splenocytes and *in vivo *infection of experimental mice, we first demonstrated that DCs are preferentially infected by MVA among various cellular components of the adaptive immune system (Fig. 1). This is consistent with our recent report of a similar DC tropism for VV infection in human PBMC [28]. Thus, despite the extensive loss of parental vaccinia host range genes from the MVA genome, the unique DC tropism of VV is well-conserved in MVA and thus may be of great biological importance for the interactions between poxviruses and the host immune system. The search for the cellular receptor(s) required for poxvirus binding and entry has continued for decades with little success. Our data suggest that the identification of poxvirus receptor(s) on DCs may greatly facilitate the design of DC-targeting Ag delivery system to further improve vaccine immunogenicity.

Infection of DCs by viruses could be either beneficial or harmful for the host immune system, depending on the subsequent phenotypic and functional changes of the infected DCs. For example, influenza virus and dengue virus infections lead to DC maturation and efficient T cell activation [34,35]. Consequently, the viruses are quickly cleared with resolution of the acute infection period. On the other hand, three viruses known to induce immunosuppression in humans, CMV, measles, and HIV, have all been documented to infect DCs directly and induce adverse functional alterations [36-39]. Our data support the contention that MVA infection efficiently induces murine DC maturation, manifested by the up-regulation of DC maturation markers (Fig. 3). We also demonstrated that following MVA infection, both BMDCs and splenic DCs secreted substantial amount of pro-inflammatory cytokine IFN-α (Fig. 4). These observations provide a rational explanation of MVA-induced immunogenicity, but are in direct contrast with the reported impairment of human DC maturation and function following VV infection [19-22,24,28]. The discrepancy could be attributed to or explained by a number of possibilities. First, it has been shown that, when viral gene expression was prevented by UV light or heat treatment, VV induced rapid human DC activation [18]. This suggests that the *de novo *synthesis of VV-encoded gene products interferes with DC maturation induced by viral binding or internalization per se. It is therefore possible that MVA infection of DCs may lead to distinct immunological consequences due to the deletion of the inhibitory genes from MVA genome. Second, MVA but not VV infection was shown to activate NF-κB in human embryonic kidney cells [40,41]. Since NF-κB activation is essential for DC activation and Ag presentation [40,41], it is possible that MVA but not VV infection activates NF-κB-dependent pathways in DCs leading to their maturation. Third, in a concurrent study, we observed rapid maturation of murine BMDCs upon VV infection (unpublished data), suggesting possible species-specific and/or DC subtype-specific differences in the consequences of poxvirus infection on DCs.

Comparative studies of the MVA and VV genomes have revealed that many VV immune evasion genes that target host cytokine and chemokine functions have been lost from the MVA genome [5,42]. This has been proposed to at least partially compensate for the inability of MVA to replicate and sustain Ag production within hosts. Here, we show that a substantial amount of IFN-α was detected in the supernatant of MVA- infected DCs (both BMDCs and splenic DCs). Interestingly, when VV was used to infect BMDCs and splenic DCs, we failed to detect any IFN-α in the supernatant [see Additional figure 1A]. Furthermore, when known amount of recombinant IFN-α was added into the supernatant samples prior to ELISA, the recombinant IFN-α was no longer detected when added into VV-infected, but not MVA-infected, DC supernatant [see Additional figure 1B]. These data are consistent with the facts that vaccinia B18R gene encodes soluble high-affinity IFN-α/β receptors and the B18R gene has been deleted from the MVA genome [43-45]. The importance of type I IFNs in the protective immunity against poxvirus infection is manifested by the attenuated virulence of B18R-knockout VV in both intranasally and intracranially infected mice [43,44]. Since type I IFN promote DC maturation and support CD8^+ ^T cell and Th1 responses [46,47], IFN-α released from MVA-infected cells may contribute to the maturation of DCs following MVA infection and the subsequent induction of innate as well as cellular immune responses.

We observed substantially reduced DC viability following MVA infection (Fig 5A). By 24 h after the infection, over 50% of DCs died. Similar to VV-induced cytopathy of human MoDC [20], we found that mature murine DCs are more resistant than immature murine DCs to MVA-induced death (Fig. 5B), and that MVA infection induced more DC apoptosis than VV infection (data not shown). The relatively higher susceptibility of DCs for MVA-induced cell death is consistent with our recent report that, compared with VV, MVA infection of human MoDCs leads to accelerated decline of intracellular anti-apoptosis genes Bcl-2 and Bcl-_L _[24]. Deletion of the anti-apoptosis gene SPI-2 from the MVA genome may also contribute to decreased DC viability following infection [24,48]. Our *in vivo *experiments provide further evidence for rapid DC apoptosis upon rMVA infection. Following intravenous infection of mice with rMVA-GFP, the number of GFP^+ ^cells in the spleen peaked at 9 h post infection and dropped sharply to background levels by 24 h post infection (data not shown). This result is in agreement with a report by Norbury et al which showed that the numbers of VV-infected cells in the draining lymph nodes following footpad injection decreased by 80% between 6 and 24 h post infection [49]. Although it is possible that the infected DCs were killed by a virus-specific immune response, as hypothesized by the authors, we think it is unlikely that naïve T cells could be called into action to kill in such a short period of time. Thus the disappearance of infected cells is more likely due to the direct cytopathic effect of the virus infection, as seen in our *in vitro *experiments.

It has been controversial whether T cell activation following poxvirus immunization is mainly due to direct Ag presentation by virus-infected DCs, rather than cross-presentation of viral Ags by uninfected DCs that have picked up the infected apoptotic cells [49]. Rapid maturation of MVA-infected DCs suggests that these DCs can directly activate T cells before they undergo apoptosis. On the other hand, substantial DC apoptosis *in vitro *and *in vivo *following MVA infection indicates that direct Ag presentation by the infected DCs is likely to diminish quickly and that cross-presentation of viral Ag by uninfected bystander DCs might be involved after the majority of the infected DCs have undergone apoptosis. In support of this hypothesis, we observed efficient phagocytosis of MVA-infected DCs by uninfected DCs (Fig. 6). Furthermore, when MVA-infected β2m^-/- ^DCs were injected into mice, Ag-specific CTL activity was detected in the spleens, although at a lower level than in mice that received MVA-infected WT DCs. Similarly, Norbury et al. has shown that β2m^-/- ^kidney cells infected with VV induced CD69 upregulation of Ag-specific CD8^+ ^T cells *in vivo *[50]. These results strongly suggest that both direct and cross-priming of CD8 T cells occur following poxvirus infection. CD8^+ ^T cell cross-priming is further supported by the identification of late poxvirus gene-derived CTL epitopes in both human and mice [51], since the inability of DCs infected by either VV or MVA to express late viral genes prevents the direct presentation of late viral Ags.

We also observed MVA infection of macrophages and B cells, although to a lesser degree comparing to DCs. It remains unclear whether these cells can also present viral Ags and activate T cells *in vivo *following MVA infection. Using the CD11c-DTR mouse model that allows the conditional ablation of DCs *in vivo*, we unambiguously demonstrated that DC depletion abrogated the generation of Ag-specific effector T cells (IFN-γ-producing). Therefore, DCs (but not other APCs) play an essential role in the generation of MVA-specific T cells *in vivo*. The preferential infection of DCs by poxviruses could serve as an efficient Ag delivery system to the most crucial APCs for the induction of strong immune responses.

## Conclusion

In summary, we present here the first systematic study of the interaction of MVA and murine DCs, using both *in vitro *and *in vivo *model systems. We demonstrate that professional APC, especially DCs, are preferentially targeted by MVA in secondary lymphoid tissues. The infected DCs express high levels of viral early Ags, undergo rapid maturation during the first 12 h post infection, produce substantial amount of IFN-α, then become apoptotic in the next 24 h. The infected DCs are readily taken up by uninfected bystander DCs, with their protein constituents then made available via cross-presentation mechanisms of antigen presentation to elicit virus-specific adaptive immune responses. DCs are required for the *in vivo *T cell activation, most likely via both direct- and cross-presentation of viral Ags. These results contribute to our understanding of the strong immunogenicity associated with the highly attenuated MVA and may be of great value for the design of safer and more effective vaccine vectors.

## Methods

### Mice

All animal work has been reviewed and approved by the Center for Animal Resources and Comparative Medicine at Emory University and Harvard Medical School. 6- to 8-week-old female BALB/c mice and C57BL/6 mice were purchased from the Jackson Laboratories (Bar Harbor, ME). MHC class I-deficient B6.129P2-*B2mtm1Unc *(β2m^-/-^) mice and CD11c-DTR-EGFP (CD11c-DTR) breeders [25] were purchased from the Jackson Laboratories and bred in a biosafety level-1 facility at Harvard Medical School. Virus-infected mice were housed in a biosafety level -2 facility.

### Virus stocks

rMVA strains were constructed to express GFP-ZEOCIN (rMVA-GFP) or lymphocytic choriomeningitis virus (LCMV) nuclear protein (NP) -ZEOCIN (rMVA-NP) [28,52] under the control of the early H5 promoter and were plaque-purified at least 5 times as previously described using standard protocol [53]. Non-recombinant MVA stock used for the construction of rMVA was kindly provided by Dr. Bernard Moss (National Institutes of Health, Bethesda, MD). MVA for the phagocytosis experiment and for the infection of CD11c-DTR mice were kindly provided by Dr. Michael S. Seaman (Beth Israel Deaconess Medical Center, Boston, MA). rMVA-GFP and rMVA-NP stocks were prepared in and titered on the chicken embryo fibroblast cell line DF-1 (gift of Dr. Harold Varmus, National Institutes of Health, Bethesda, MD). Viruses were concentrated by ultracentrifugation over 36% (weight/volume) sucrose, and titers were determined using standard plaque assays. LCMV stocks (Armstrong strain) were kindly provided by Dr. Rafi Ahmed (Emory Vaccine Research Center, Atlanta, GA).

### Cells

DF-1 and HeLa cells are described above. These cell lines were maintained in complete DMEM supplemented with 10% FBS, 2 mM L-glutamine and antibiotics. EL4 cells (H-2^b^) (ATCC) were maintained in RPMI supplemented with 10% FBS, 2 mM L-glutamine and antibiotics. Splenic DCs were isolated from the spleens of BALB/c mice that received daily injections of 20 μg of recombinant flt3L-IgG_2 _for 9 days [54,55]. Briefly, spleen fragments were digested with 1 μg/ml collagenase (Worthington, Lakewood, NJ) for 30 min, followed by erythrocyte depletion with RBC lysing buffer (Sigma-Aldrich Co., St. Louis, MO). DCs were then isolated from this single cell suspension using CD11c magnetic beads (Miltenyi Biotec, Auburn, CA) according to the manufacturer's instructions, and the purity of DCs was greater than 90% as evaluated by flow cytometry (data not shown).

Bone marrow-derived DCs (BMDCs) were generated from mouse bone marrow cultures as described previously with minor modifications [56]. Briefly, bone marrow was flushed from femurs and tibias and depleted of erythrocytes with RBC lysing buffer. Cells were subsequently plated on 6-well tissue culture plates at 4 × 10^6^/well in 3 ml complete RPMI 1640 medium (CM, contains 10% FBS, 2 mM L-glutamine, and 10 μg/ml gentamicin sulfate), incubated at 37°C for 3 h, and then the non-adherent cells were removed. CM containing 10 ng/ml rmGM-CSF and 3 ng/ml rmIL-4 (both from R&D Systems, Minneapolis, MN) was added at 3 ml per well and the plates were incubated at 37°C for 1 week. Cultures were fed by gently aspirating 2 ml/well of medium and adding back fresh CM containing cytokines on days 2, 4 and 6 of the culture. When mature DCs were required, 10 μg/ml LPS (Sigma-Aldrich Co.) was added to the culture on day 6 for 24 h. On day 7, non-adherent cells were harvested, and the purity of the DCs culture was evaluated by cell surface staining with fluorescence-conjugated mAbs to CD11b (M1/70), CD11c (HL3), CD3 (145-2C11), and CD45R/B220 (RA3-6B2) (BD Biosciences Pharmingen, San Diego, CA). Approximally 70–90% of the cells were CD11c^+^, as determined by flow cytometry (data not shown).

### Viral infection

For *in vitro *infections, cells were resuspended at 1 × 10^7^/ml CM in 15 ml polypropylene tubes (Becton Dickinson Labware). An appropriate volume of the indicated virus stock was added to reach the multiplicity of infection (MOI) required for each experiment. Following 1 h-incubation at 37°C, cells were washed three times with medium, resuspended at 1 × 10^6^/ml in CM on 24-well plates and cultured at 37°C until harvesting. Mock-treated cells were manipulated in the same way except that no virus was added. For *in vivo *i.v. infection, BALB/c mice were i.v. injected with 15 μg DNA plasmid pNGVL3-hFLex [57] (obtained from National Gene Vector Laboratory, University of Michigan, and provided by Dr. Abdul M. Jabbar, Emory Vaccine Center) in 1.6 ml PBS to expand DC population *in vivo*. Five days later, mice were infected i.v. with 3 × 10^8 ^plaque forming units (PFU) of rMVA-GFP in 0.5 ml PBS. Uninfected mice were injected with 0.5 ml PBS as controls. For intraperitoneal (i.p.) infection, CD11c-DTR mice and wild type (WT) littermates were infected with 2 × 10^6 ^PFU MVA.

### Cell surface staining and flow cytometry

At the indicated time points post viral infection, splenocytes were stained with mAbs against CD3, B220, CD11b, CD11c, CD8a (Ly-2) (53-6.7), and CD4 (L3T4) (RM4-5) (BD Biosciences Pharmingen). At the indicated time points following *in vitro *infection, DF-1 cells, BMDCs, and splenic DCs were stained with purified anti-vaccinia late surface protein A56R using mAb VV1-4G9 (kindly provided by Dr. Alan Schmaljohn, USAMRIID, Frederick, MD) and secondary PE-conjugated goat anti-mouse IgG (Biosource International, Camarillo, CA). BMDCs and splenic DCs were also stained with mAbs against DC maturation markers CD80 (16-10A1), CD86 (GL1), CD40 (5C3), and I-A^d ^(AMS-32.1) (BD Biosciences Pharmingen). All staining steps were performed by incubating cells and Abs at 4°C for 20 min followed by three washes with staining buffer. Samples were then processed using a FACSCalibur flow cytometer (Becton Dickinson Labware) and the data were analyzed using Flowjo software (Tree Star, Inc., Ashland, OR).

### Quantitation of IFN-α production by ELISA

BMDCs or splenic DCs were mock- treated or infected with rMVA-GFP. Supernatant samples were harvested at 10 or 24 h following the treatment and stored at -80°C if not used immediately. IFN-α in the supernatant was detected using a commercial colorimetric sandwich ELISA kit (R&D Systems) according to manufacturer's instructions.

### Viability study

Viability of BMDCs was determined by trypan blue exclusion at various time points following infection.

### Phagocytosis assay

BMDCs were labeled with the green fluorescent dye PKH-67 (Sigma-Aldrich Co.) according to the manufacturer's instructions and infected with MVA at a MOI of 10 for 18 h. They were then mixed with uninfected immature BMDCs labeled with the red fluorescent dye PKH-26 (Sigma-Aldrich Co.) for 4 h at either 37°C or 4°C. Phagocytosis of infected DCs by uninfected DCs (PKH-67^+ ^PKH-26^+ ^population) was visualized by flow cytometry.

### BMDC immunization and detection of cytotoxic T lymphocyte (CTL) activity

BMDCs derived from either WT C57BL/6 mice (B6.DCs) or β2m^-/-^mice (β2m^-/-^DCs) were infected with rMVA-NP or control rMVA-GFP at a MOI of 10 for 6 h. The cells were then extensively washed with PBS and UV-irradiated to inactivate any residual viruses and prevent co-injection of infectious MVA. They were then injected (1 × 10^6^) into B6 mice that had been i.p.-infected with 2 × 10^5 ^PFU LCMV 30 days earlier. Five days after the DC injection, spleens were harvested and NP_396–404_-specific CTL activity was assessed by fluorescent cellular cytotoxicity assay as previously described [58]. Briefly, target EL4 cells were incubated at 37°C 5% CO_2 _for 1 h in the presence of 1 μM NP_396–404 _peptide and 3 μM cell tracker orange (CTO, Invitrogen, Carlsbad, California). Cells were washed once with PBS and resuspended in CM at 1 × 10^6^/ml. Target cells and effector splenocytes from BMDC-immunized mice (100 μl) were co-cultured on 96-well U-bottom plates for 4 h at 37°C 5% CO_2_. The cells were then pelleted and incubated in 75 μl/well fluorogenic caspase 3 substrate R2D2 (10 μM, kindly provided by Dr. Beverly Packard, OncoImmunin Inc., Gaithersburg, MD) for 30 min followed by two washes with cold PBS. Sample acquisition and analysis are as described above. The cleaved caspase substrate has the following fluorescence peak characteristics: λ_ex _= 505 nm and λ_em _= 530 nm, and can be detected in the FL1 channel. CTO can be detected in the FL2 channel. The percentage of specific killing = [% caspase^+ ^CTO^+ ^cells/(% caspase^+ ^CTO^+ ^cells + % caspase^- ^CTO^+ ^cells)] × 100%.

#### *In vivo *DC depletion and intracellular IFN-γ staining

Age and gender-matched CD11c-DTR mice and WT litter-mates were injected i.p. with 12 ng/g body weight of diphtheria toxin (DT) (List Biological Laboratories, Campbell, CA) on day 0 and day 2 [25,26]. Six hours after the first DT injection, mice were infected i.p. with MVA at 5 × 10^6 ^PFU. Spleens were harvested on day 6 following the infection. Efficiency of DC depletion was determined by flow cytometry. Single cell suspensions (effector cells) were incubated with MVA-infected naïve splenocytes (target cells) in 96-well U-bottom plate (10^6^/well effector and target cells each) in the presence of GolgiStop (BD Bioscience) for 12 h. Cells were then blocked with anti-CD16/CD32 FcγR III/II (2.4G2) and subjected to surface staining with anti-CD3 and CD8, as described above. Subsequent intracellular staining was performed using anti-mouse IFN-γ (XMG1.2), rat-IgG1 isotype control (R3-34), and CytoFix/CytoPerm kit (BD Biosciences) according to manufacturer's instructions.

### Statistical analysis

All statistical analyses were made with Microsoft Excel software (Microsoft). Statistical comparisons between data sets were made with a two-tailed homoscedastic Student's t-test.

## List of abbreviations

VV: vaccinia virus; MVA: Modified vaccinia Ankara; DC: dendritic cell; MoDC: monocyte-derived DC; DTR: diphtheria toxin receptor; LCMV: lymphocytic choriomeningitis virus; NP: nuclear protein; BMDC: bone marrow derived DC; CM: complete medium; MOI: multiplicity of infection; PFU: plaque forming unit; i.p., intraperitoneal; WT: wild type; CTL: cytotoxic T lymphocyte; CTO: cell tracker orange; DT: diphtheria toxin.

## Authors' contributions

LL designed, performed and analyzed all the experiments, wrote the manuscript. RC created the rMVA-GFP and rMVA-NP, and generated data in Fig. 2B. MBF participated in the design of the experiment and interpretation of data, revised the manuscript critically for important intellectual content. All authors read and approved the final manuscript.

## Supplementary Material

Additional file 1 **IFN-α was not detected in the supernatant of VV-infected DCs**. (A) Immature BMDCs or purified CD11c^+ ^splenic DCs were either mock treated or infected with rVV-EGFP (a kind gift of Dr. Lawrence Corey, University of Washington, Seattle, WA) at a MOI of 10. Supernatant samples were collected at 10 and 24 h post infection, and IFN-α levels were measured by ELISA. These data are representative of three independent experiments. (B) VV-infected BMDCs produced a soluble factor(s) that abrogated the detection of IFN-α in the supernatant. Immature BMDCs were infected with rMVA-GFP or rVV-EGFP at a MOI of 10. Supernatant samples were collected at 24 h post infection. Various amounts of rmIFN-α were added into supernatant samples and ELISA was subsequently performed to detect IFN-α. Click here for file
