# Supplementary figures and images for: Dendritic Cells are preferentially targeted among hematolymphocytes by Modified Vaccinia Virus Ankara and play a key role in the induction of virus-specific T cell responses in vivo
Source: BMC Immunol. 2008 Apr 15;9:15. doi: 10.1186/1471-2172-9-15 (PMC2359732; doi:10.1186/1471-2172-9-15)

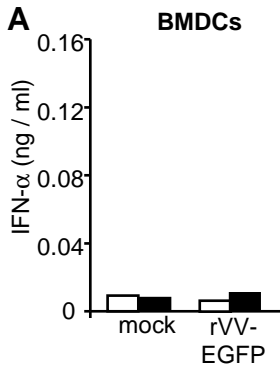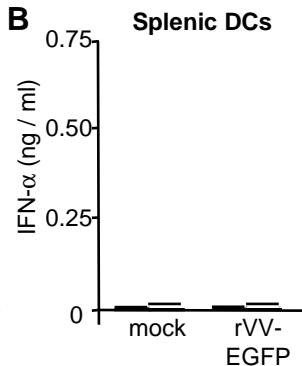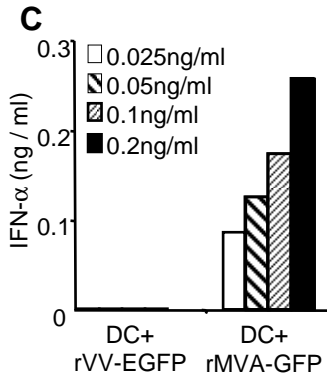

Supplement: Additional file 1 — IFN-α was not detected in the supernatant of VV-infected DCs. (A) Immature BMDCs or purified CD11c+ splenic DCs were either mock treated or infected with rVV-EGFP (a kind gift of Dr. Lawrence Corey, University of Washington, Seattle, WA) at a MOI of 10. Supernatant samples were collected at 10 and 24 h post infection, and IFN-α levels were measured by ELISA. These data are representative of three independent experiments. (B) VV-infected BMDCs produced a soluble factor(s) that abrogated the detection of IFN-α in the supernatant. Immature BMDCs were infected with rMVA-GFP or rVV-EGFP at a MOI of 10. Supernatant samples were collected at 24 h post infection. Various amounts of rmIFN-α were added into supernatant samples and ELISA was subsequently performed to detect IFN-α. [file 1471-2172-9-15-S1.pdf]
